# Serotonin‐Affecting Antidepressant Use in Relation to Platelet Reactivity

**DOI:** 10.1002/cpt.2517

**Published:** 2022-01-10

**Authors:** Joseph Grech, Melissa Victoria Chan, Chinedu Ochin, Amber Lachapelle, Florian Thibord, Zoe Schneider, Bongani Brian Nkambule, Paul Charles John Armstrong, Catherine Wallace de Melendez, Katherine L. Tucker, Mahdi Garelnabi, Timothy David Warner, Ming‐Huei Chen, Andrew Danner Johnson

**Affiliations:** ^1^ Population Sciences Branch National Heart, Lung, and Blood Institute Framingham Massachusetts USA; ^2^ The Blizard Institute London UK; ^3^ Department of Biomedical and Nutritional Sciences University of Massachusetts Lowell Lowell Massachusetts USA; ^4^ Center for Population Health University of Massachusetts Lowell Lowell Massachusetts USA

## Abstract

Depression is an independent risk factor of cardiovascular disease morbidity. Serotonin is a key neurotransmitter in depressive pathology, contained within platelets, and is a weak activator of platelets. Our study assessed the link between platelet reactivity traits, depression, and antidepressant (AD) use in a large population sample. Our study was conducted in the Framingham Heart Study (*n *= 3,140), and AD use (*n *= 563) and aspirin use (*n *= 681) were noted. Depression was measured using the Center for Epidemiological Studies‐Depression (CES‐D) survey. Platelet reactivity traits were measured across multiple agonists using five distinct assays. We utilized a linear mixed effects model to test associations between platelet traits and depression, adjusting for age, sex, aspirin use, and AD use. Similarly, we analyzed trait associations with any AD use, serotonin‐affecting ADs, and norepinephrine‐affecting ADs, respectively. There were strong associations with reduced platelet function and AD use, particularly with serotonin‐affecting medications. This included lower Optimul epinephrine maximal aggregation (*P* = 4.87E‐13), higher U46619 half maximal effective concentration (*P* = 9.09E‐11), lower light transmission aggregometry (LTA) adenosine diphosphate (ADP) final aggregation (*P* = 1.03E‐05), and higher LTA ADP disaggregation (*P* = 2.28E‐05). We found similar associations with serotonin‐affecting ADs in an aspirin‐taking subset of our sample. There were no significant associations between platelet traits and depression. In the largest study yet of AD use and platelet function we show that antidepressants, particularly serotonin‐affecting ADs, inhibit platelets. We did not find evidence that depressive symptomatology in the absence of medication is associated with altered platelet function. Our results are consistent with AD use leading to platelet serotonin depletions, decreased stability of platelet aggregates, and overall decreased aggregation to multiple agonists, which may be a mechanism by which ADs increase risk of bleeding and decrease risk of thrombosis.

**Study Highlights**

**WHAT IS THE CURRENT KNOWLEDGE ON THE TOPIC?**

☑ Past research has shown positive associations between depression and platelet reactivity, and negative associations between antidepressant use and platelet reactivity. These studies, however, have predominantly been performed in small sample sizes and have utilized heterogenous platelet function assays, leading to inconsistent results.

**WHAT QUESTION DID THIS STUDY ADDRESS?**

☑ Our study looked at the effects of depression and antidepressant use, respectively, on platelet reactivity using five distinct platelet function assays across multiple agonists in a large sample size.

**WHAT DOES THIS STUDY ADD TO OUR KNOWLEDGE?**

☑ Our study shows that antidepressants, particularly those affecting the serotonergic system, have a clear inhibitory effect on platelets in a large, predominantly healthy population. These results spanned multiple platelet reactivity traits derived from a range of platelet agonists. The strongest result of serotonin drugs associating with decreased thromboxane receptor activation was replicated in a distinct Hispanic population. We also show significant associations in an aspirin‐taking subset indicating a synergistic platelet inhibitory effect with concurrent antidepressant and aspirin use. Future researchers employing platelet assays should account for antidepressant use as well as other antiplatelet medications.

**HOW MIGHT THIS CHANGE CLINICAL PHARMACOLOGY OR TRANSLATIONAL SCIENCE?**

☑ Our results indicate that the antiplatelet effect of serotonin‐affecting antidepressants may increase the risk of adverse bleeding or decrease the risk of thrombosis. These results may inform future prospective studies of antidepressant use and bleeding, and thrombotic events, respectively, and may ultimately suggest platelet function testing in clinic for prescription and monitoring of antidepressants.

Previous studies have cited associations between depression and cardiovascular disease (CVD) mortality and morbidity.^1^, ^2^, ^3^ Additionally, comorbidity of CVD and depression is commonly seen in clinical settings and is linked to poor prognosis.^3^ Given that impaired central serotonin signaling has been linked to depression, and that serotonin can induce platelet aggregability and is released from platelet dense granules upon activation, platelets have been hypothesized as a link between CVD and depression.^1^ Past studies in the field have utilized a wide range of platelet activation and aggregation assays in small sample sizes, with variable findings.^1^, ^4^, ^5^, ^6^, ^7^, ^8^, ^9^, ^10^, ^11^, ^12^ For example, some studies found that depression was associated with increased expression of activated platelet markers such as glycoprotein IIb/IIIa and P‐selectin, hyper‐aggregation in response to collagen and thrombin, and increased serotonin‐augmented epinephrine aggregation.^5^, ^8^, ^9^, ^13^ In other cases, however, no relationships were found.^11^ To better understand the role of platelets in linking depression with CVD, a large‐scale study with multiple platelet reactivity parameters across a breadth of agonists is warranted.

Antidepressants (ADs), particularly selective serotonin reuptake inhibitors (SSRIs), are often prescribed after a diagnosis of depression or other affective disorders.^14^ As serotonin transporters are similarly expressed in platelets and the central nervous system, some classes of antidepressants potentially impact platelet function.^15^ Several studies have sought to elucidate the effects of different antidepressant drug classes on platelet function and have subsequently shown that serotonin‐affecting ADs exert an inhibitory effect on platelets across a broad range of platelet function assays and agonists.^14^, ^15^, ^16^ However, given the heterogeneity of platelet assays and small sample sizes utilized, the extent to which these drugs affect platelets remains inconclusive.

We aimed to assess relationships between depressive symptomatology and AD use, respectively, and platelet reactivity traits from five distinct platelet function assays and a broad range of platelet agonists in a large sample within the Framingham Heart Study (FHS). We hypothesized that depression would be associated with higher, and AD use lower, platelet reactivity trait values.

## METHODS

### Study sample

**Figure 1:**
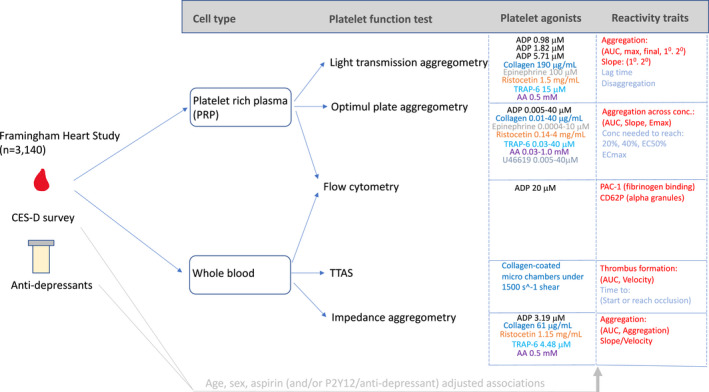
Study overview and design. Platelet agonists are consistently color coded across assay platforms. Platelet reactivity trait readouts are coded by color as follows: red indicates as the trait increases platelet reactivity increases; blue indicates as the trait increases platelet reactivity decreases. Further information on methods and details are given in **Table** **S1** and the **Supplementary Text**. AA, arachidonic acid; ADP, adenosine diphosphate; AUC, area under the curve; CES‐D, Center for Epidemiological Studies‐Depression [survey]; Conc., concentration; Ec_max_, effective concentration needed to reach maximal aggregation; EC_50_, half maximal effective concentration; E_max_, maximal aggregation observed; PAC‐1, antibody specific for platelet fibriogen receptor; PRP, platelet‐rich plasma; TRAP‐6, thrombin receptor activating peptide‐6; T‐TAS, Total Thrombus formation Analysis System. [Colour figure can be viewed at wileyonlinelibrary.com]

The overall study design is shown in **Figure** **1** and a guide to the description of the platelet traits and their interpretation is shown in **Table** **S1**. The FHS is a community‐based cohort study. We included participants from Generation Three and New Offspring Spouse (NOS) cohorts, at Examination Three (years: 2016–2019) (**Table** **1**). These participants are largely of European ancestry. Other characteristics of these cohorts are previously described.^17^, ^18^ All participants provided written informed consent, and the Boston University Medical Center Institutional Review Board (IRB) approved the study protocol.

**Table 1:** Demographics of main study sample

Generation 3 / NOS cohorts at exam 3		
Baseline characteristic	Mean/n	SD/%
Age (y)	54.5	8.97
Women (n/%)	1,682	53.6
BMI (kg/m^2^)	28.6	5.90
SBP (mmHG)	120	14.1
DBP (mmHG)	75.7	8.64
Fasting blood glucose (mg/dL)	100	21.5
Triglycerides	113	80.6
Total cholesterol (mg/dL)	190	36.2
HDL cholesterol (mg/dL)	59.9	19.6
Diabetes mellitus (*n*/%)	272	8.66
Hypertension (*n*/%)	1,810	52.8
Smoker in past year	227	7.24
Aspirin use (*n*/%)	681	21.7
Alcohol intake (units of beer/wk)	2.12	5.01
Alcohol intake (units of wine/wk)	2.08	3.72
Alcohol intake (units of liquor/wk)	1.08	3.07

Diabetes mellitus includes individuals with a fasting blood glucose ≥ 126, a nonfasting blood glucose ≥ 200, or antidiabetic medication self‐report. Hypertension includes individuals with SBP ≥ 130 or DBP ≥ 80 or antihypertensive medication self‐report. Smoker includes individuals reporting that they smoked regularly in the past year. Aspirin use defined by light transmission aggregometry (LTA) or multiplate (MP) impedance aggregometry assay cutoffs using cyclooxygenase‐1 (COX‐1)–specific agonist (1.6 mM arachidonic acid), or self‐report if previous assays were unavailable.

BMI, body mass index; DBP, diastolic blood pressure; HDL, high‐density lipoprotein; NOS, New Offspring Spouse; SBP, systolic blood pressure.

### Replication sample

The Boston Puerto Rican Health Study (BPRHS) is a longitudinal cohort study of Puerto Rican adults residing in greater Boston, Massachusetts area (demographics in **Table** **S2**). Data for the current study are from the fourth wave of data collection (2018–2021, *n *= 550). The study design, enrollment criteria, and other characteristics have been published previously.^19^ All participants provided written informed consent, and the IRBs at both Tufts University and University of Massachusetts Lowell approved the study protocol.

### Depressive symptomology

We quantified depressive symptomology using the Center for Epidemiological Studies Depression (CES‐D) scale. Briefly, the CES‐D is a self‐report survey consisting of 20 questions, answered on a Likert scale, relating to depressive mood.^20^ The survey is scored on a range of 0 to 60.

Past studies have established the CES‐D as a valid method of analyzing depressive symptoms in epidemiological studies, including with Latino populations.^19^, ^20^, ^21^ Scoring guidelines suggest that an individual scoring ≥16 is at risk for clinical depression, and this threshold was used to categorize “depression.” Similarly, we analyzed “severe depression” as a those scoring ≥21.

### Aspirin use

Light transmission aggregometry (LTA) with arachidonic acid (AA) agonist stimulation is a highly sensitive means of detecting aspirin use, as aspirin takers generally do not reach >20% final aggregation on the LTA‐AA assay, and nonaspirin takers generally do not score <60% final aggregation.^22^ Thus, we utilized a liberal threshold of 40% final aggregation on the LTA‐AA assay to define aspirin use in our study (**Figure** **S1**). Where individual LTA‐AA participant data were unavailable, we utilized the Multiplate AA/Aspi assay at the manufacturer recommended cutoff of 40 area under the time‐aggregation curve (AUC) aggregation for defining aspirin use, or self‐report. Only self‐report of regular (daily or weekly) aspirin use was available for the BPRHS. Many past studies have implicated aspirin as having a major effect on platelet assays. In models of aspirin effects alone, assays dependent upon endogenous generation, such as AA or low concentrations of ADP, are strongly suppressed by aspirin use; activators such as TRAP‐6 (thrombin receptor activating peptide‐6), and high concentrations of agonists like ADP, or the thromboxane A2–mimetic U46619 are less affected (**Table** **S3**). Because of these effects, we adjusted for aspirin use in our analyses.

### Antidepressant use

AD use was determined by participant self‐report in both cohorts. Participants were advised to bring their medicines to their examination, and these medications were mapped to corresponding Anatomical Therapeutic Chemical codes. Programming code (**Supplemental Text**) pattern‐matched drug names to capture all potential ADs. To capture broad and specific effects of ADs, we analyzed associations between platelet reactivity traits and any AD use, serotonin‐affecting AD use, SSRI use, norepinephrine and dopamine reuptake inhibitor (NDRI) use, and norepinephrine‐affecting AD use, respectively. In instances where a participant was on a drug affecting both serotonin and norepinephrine reuptake, participants were included in both serotonin‐affecting and norepinephrine‐affecting classes. Similarly, participants on multiple ADs were included in all eligible classes. After observing significant associations in the serotonin‐affecting medication classes, we conducted subanalyses with further adjustment for platelet ADP P2Y12 receptor (P2Y12) inhibitor medication use. We focused on the most prevalent medications that specifically target serotonin through different mechanisms: the SSRIs that target serotonin reuptake (*n *= 362) and the serotonin antagonist and reuptake inhibitors (SARIs) that target reuptake and 5HT2A/2C receptors (trazodone *n *= 49 / nefazodone *n *= 1).

### Blood collection and platelet assays

Briefly, fasting blood collection was taken in the morning and either whole blood or isolated platelet‐rich plasma was used in multiple platelet function testing approaches with varied agonists and concentrations that target specific pathways of activation. Platelet assay methods are described in detail in the **Supplemental Text**. **Figure** **1** provides an overview of the assays and **Table** **S1** provides specific details of each assay.

### Statistical analysis

We analyzed associations between platelet traits and CES‐D ≥16, CES‐D ≥21, any AD use, SSRI AD use, SARI AD use, serotonin‐affecting AD use, norepinephrine‐affecting AD use, and NDRI AD use, respectively. To do so, we applied an inverse normal transformation to residuals obtained from linear regression of platelet traits adjusting for age, sex, and aspirin use. Given that many individuals with depression use ADs (**Table** **2**), regression analyses for CES‐D16/21 additionally adjusted for AD use. For Optimul assay traits, UK/FHS plate source was also adjusted for in regression analyses. A linear mixed effects model implemented in *lmekin* function of *coxme* R package (https://cran.r‐project.org/web/packages/coxme/index.html), which can account for familial correlation, was used for association testing between transformed residuals and depression/AD use categories. We secondarily tested associations between platelet traits and CES‐D16/21 in non‐AD users. Sex‐stratified and sex‐interaction analyses were conducted to assess the effect on response to ADs, except that in these analyses sex was not adjusted for in the regression analysis for obtaining residuals. We applied a multiple test correction threshold for significance of *P *< 5.55E‐4, based on principal components analyses to determine independent traits. Further details of the threshold derivation are given in the **Supplemental Text**.

**Table 2:** Sample sizes for depression and AD variables

Generation 3 / NOS Cohort at Exam 3 (*n *= 3,140)						
	Total sample		Men (*n *= 1,458)		Women (*n *= 1,682)	
Baseline characteristic	*n*	%	*n*	%	*n*	%
CES‐D ≥ 16	296	9.43	110	7.54	186	11.1
CES‐D ≥ 21	159	5.06	51	3.50	108	6.42
AD users	563	17.9	157	10.8	406	24.1
Single AD users	482	15.5	136	9.32	346	20.6
Two AD users	75	2.39	17	1.17	58	3.45
Three AD users	6	0.19	4	0.27	2	0.11
Serotonin‐affecting AD users	508	16.2	133	9.12	375	22.3
SSRI AD users	361	11.5	91	6.24	270	16.1
SARI AD users						
Norepinephrine‐affecting AD users	215	6.85	64	4.39	151	8.98
NDRI users	94	2.99	35	2.40	59	3.51

CES‐D score	On/Off ADs	% on	On/Off ADs	% on	On/Off ADs	% on
CES‐D ≥ 16	138/158	46.6	41/69	37.3	97/89	52.2
CES‐D ≥ 21	87/72	54.7	19/32	37.3	68/40	63.0

AD, antidepressant; CES‐D, Center for Epidemiological Studies‐Depression [survey]; NDRI, norepinephrine and dopamine reuptake inhibitor; NOS, New Offspring Spouse; SARI, serotonin antagonist and reuptake inhibitor; SSRI, selective serotonin reuptake inhibitor.

### Aspirin use strata and serotonin‐affecting drugs

To observe the antiplatelet effects of concurrent aspirin and serotonin‐affecting AD use, we conducted secondary regression analyses in the subset of our cohort taking aspirin and serotonin‐affecting ADs, focusing on the 26 platelet traits that reached statistical significance in our primary analyses. The statistical method was identical to the main analyses outlined above; strict Bonferroni correction of the 26 traits gave a statistical significance threshold of *P *< 0.00192.

### Boston Puerto Rican Health Study serotonin‐affecting antidepressant replication analysis

Given the strong associations between serotonin‐affecting medication and U46619 Optimul and consistent findings within the aspirin‐taking subgroup, we sought replication of FHS results in the BPRHS. This subanalysis included 394 individuals with U46619 Optimul aggregation data (including *n *= 133 on serotonin‐affecting ADs), and an aspirin‐takers subpopulation comprised of 184 individuals (*n *= 62 on serotonin‐affecting ADs). Application of Bonferroni correction to our five U46619 traits yielded a significance threshold of *P* < 0.01.

## RESULTS

### Study sample

Our FHS sample consisted of 3,140 participants with mean age of 54.5 years and was 53.5% female; 681 participants met inclusion for aspirin use. Our BPRHS sample consisted of 394 participants with mean age of 68.1 years and was 77.2% female; 46.7% reported using aspirin. Sample sizes for each assay varied based on instrument and blood collection availability throughout the exam period. Exact sample sizes for each trait can be found in **Tables** **S4–S15**. Characteristics of all participants in FHS can be found in **Table** **1**, and for BPRHS in **Table** **S2**.

### Depressive symptoms and AD use in the FHS

Of the 3,140 participants included in the study, 296 (9.43%) scored ≥16 on the CES‐D, 159 participants (5.06%) scored ≥21, and 563 participants (17.9%) reported AD use. A breakdown of AD use and CES‐D score characteristics is shown in **Table** **2**, and sample sizes for specific ADs used by participants are given in **Table** **3**.

**Table 3:** Sample sizes for individual medications used within each AD class

Antidepressant drugs in the FHS		
Serotonin‐affecting	Norepinephrine‐affecting	Serotonin‐affecting and norepinephrine‐affecting
**SSRIs** Sertraline: 99 Fluoxetine: 96 Citalopram: 78 Escitalopram: 61 Paroxetine: 24 Fluvoxamine: 2 Vilazodone: 2 **SARIs** Trazodone: 49 Nefazodone: 1	**NDRI** Bupropion: 94 **TCA** Desipramine: 5	**TCAs** ^a^ Amitriptyline: 34 Nortriptyline: 11 Doxepin: 3 Imipramine: 3 Protriptyline: 1 **SNRIs** Venlafaxine: 39 Duloxetine: 32 Desvenlafaxine: 5
**SMS** Vortioxetine: 3 **TCA** Mirtazapine: 7		

AD, antidepressant; FHS, Framingham Heart Study; NDRI, norepinephrine and dopamine reuptake inhibitor; SARIs, serotonin antagonist and reuptake inhibitor; SMS, serotonin modulator and stimulator; SNRIs, serotonin and norepinephrine reuptake inhibitor; SSRIs, selective serotonin reuptake inhibitors; TCA, tricyclic antidepressant.

^a^ A search for the TCA clomipramine (and synonyms) was conducted but there were no individuals reported to be taking this medication. This may be due to more limited US Food and Drug Administration (FDA) approvals for indications in the United States for clomipramine compared with other countries.

### AD use and platelet traits

We observed 21 platelet reactivity traits associated with AD use (**Table** **4**, **Table** **S4**), predominantly suggesting AD use having an inhibitory effect on platelets, and appearing mostly driven by serotonin‐affecting medications (e.g., epinephrine Optimul AUC beta = −0.31, *P *= 5.70E‐10, U46619 Optimul AUC beta = −0.32, *P *= 1.83E‐10). Analysis of SSRIs alone was concordant with the serotonin‐affecting AD class. However, the greater sample size of serotonin‐affecting ADs, due to inclusion of SARIs, and serotonin and norepinephrine reuptake inhibitors and tricyclic antidepressants, may be a reason for the more significant associations. Of note, we found strong associations between ADP (middle concentration, 1.82 μM) disaggregation (beta = 0.207, *P *= 2.28E‐05) and final aggregation (beta = −0.215, *P *= 1.03E‐05) in LTA, suggesting an attenuated aggregation response and less stable formation of platelet aggregates after ADP stimulation. Additionally, we observed associations for and secondary aggregation (beta = −0.248, *P *= 1.00E‐06) and slope (beta = −0.193, *P *= 1.21E‐04) to ADP (low concentration, 0.95 μM), indicating an attenuation in secondary amplification. Serotonin‐affecting AD use and SSRI use results can be found in **Table** **S5** and **Table** **S6**, respectively. Sex‐specific associations in serotonin‐affecting medications classes were often more significant in women than men, which may partially reflect much higher AD medication use rates (**Table** **2**) in women, but interestingly there were no significant sex‐interactions since the effect estimates in men were all similar.

**Table 4:** Significant results for platelet traits passing multiple correction

Platelet Trait	Assay	*N*	Any AD use		*P* value	Serotonin‐affecting ADs		*P* value	SSRI use		*P* value	Norepinephrine‐affecting ADs		*P* value	NDRI use		*P* value
			Beta	SE		Beta	SE		Beta	SE		Beta	SE		Beta	SE	
AA EC_max_	Optimul	2587	**0.23**	**0.05**	**4.94E‐06**	**0.24**	**0.05**	**3.53E‐06**	**0.23**	**0.06**	**2.03E‐04**	0.19	0.08	0.013	0.14	0.11	0.224
ADP Agg20	Optimul	2735	**0.19**	**0.05**	**1.08E‐04**	**0.19**	**0.05**	**2.07E‐04**	**0.23**	**0.06**	**1.27E‐04**	0.10	0.08	0.182	0.03	0.12	0.822
ADP Agg40	Optimul	2770	**0.19**	**0.05**	**1.48E‐04**	**0.20**	**0.05**	**1.05E‐04**	0.18	0.06	1.94E‐03	0.13	0.08	0.080	0.13	0.11	0.260
ADP EC_50_	Optimul	2775	**0.19**	**0.05**	**1.54E‐04**	**0.20**	**0.05**	**1.38E‐04**	0.17	0.06	3.32E‐03	0.14	0.07	0.066	0.11	0.11	0.325
ADP low final agg	LTA	2853	−**0.17**	**0.05**	**5.34E‐04**	−**0.18**	**0.05**	**3.86E‐04**	−0.15	0.06	7.95E‐03	−0.19	0.07	9.02E‐03	−0.12	0.10	0.253
ADP low 2^0^ agg	LTA	2853	−**0.23**	**0.05**	**1.55E‐06**	−**0.25**	**0.05**	**1.00E‐06**	−**0.23**	**0.06**	**7.40E‐05**	−0.20	0.07	6.33E‐03	−0.08	0.11	0.465
ADP low 2^0^ slope	LTA	2853	−**0.18**	**0.05**	**2.45E‐04**	−**0.19**	**0.05**	**1.21E‐04**	−**0.25**	**0.06**	**1.27E‐05**	−0.07	0.07	0.301	−0.04	0.10	0.701
ADP mid disagg	LTA	3050	**0.20**	**0.05**	**1.65E‐05**	**0.21**	**0.05**	**2.28E‐05**	0.16	0.06	5.63E‐03	**0.24**	**0.07**	**5.47E‐04**	0.27	0.10	0.010
ADP mid final agg	LTA	3050	−**0.20**	**0.05**	**2.42E‐05**	−**0.21**	**0.05**	**1.03E‐05**	−0.15	0.06	9.35E‐03	−**0.24**	**0.07**	**4.96E‐04**	−0.16	0.10	0.115
ADP mid max agg	LTA	3050	−0.16	0.05	7.38E‐04	−**0.17**	**0.05**	**4.48E‐04**	−0.10	0.06	0.080	−0.20	0.07	4.98E‐03	−0.09	0.10	0.362
ADP velocity	MP	3125	0.05	0.05	0.263	0.03	0.05	0.573	0.08	0.05	0.170	0.10	0.07	0.161	**0.35**	**0.10**	**4.87E‐04**
Collagen Agg20	Optimul	2700	0.17	0.05	6.39E‐04	**0.24**	**0.05**	**6.14E‐06**	0.19	0.06	1.65E‐03	0.13	0.08	0.083	−0.07	0.11	0.514
Collagen Agg40	Optimul	2738	**0.21**	**0.05**	**1.42E‐05**	**0.27**	**0.05**	**1.76E‐07**	**0.26**	**0.06**	**1.10E‐05**	0.11	0.07	0.124	−0.08	0.11	0.455
Collagen AUC	Optimul	2757	−0.17	0.05	7.12E‐04	−**0.22**	**0.05**	**2.64E‐05**	−0.17	0.06	4.34E‐03	−0.15	0.07	0.047	−0.02	0.11	0.880
Collagen EC_50_	Optimul	2742	0.17	0.05	7.51E‐04	**0.20**	**0.05**	**1.14E‐04**	0.17	0.06	4.69E‐03	0.16	0.07	0.027	0.03	0.11	0.781
Collagen EC_max_	Optimul	2757	**0.18**	**0.05**	**2.95E‐04**	**0.18**	**0.05**	**3.74E‐04**	**0.26**	**0.06**	**1.83E‐05**	0.02	0.07	0.835	−0.03	0.11	0.784
Epinephrine Agg20	Optimul	2749	**0.28**	**0.05**	**1.55E‐08**	**0.29**	**0.05**	**1.59E‐08**	**0.26**	**0.06**	**1.27E‐05**	**0.32**	**0.07**	**2.02E‐05**	0.26	0.11	0.016
Epinephrine AUC	Optimul	2799	−**0.30**	**0.05**	**9.04E‐10**	−**0.31**	**0.05**	**5.70E‐10**	−**0.30**	**0.06**	**2.70E‐07**	−0.25	0.07	7.80E‐04	−0.20	0.11	0.066
Epinephrine EC_50_	Optimul	2777	**0.28**	**0.05**	**8.32E‐09**	**0.28**	**0.05**	**2.85E‐08**	**0.26**	**0.06**	**1.09E‐05**	**0.28**	**0.07**	**1.10E‐04**	0.25	0.11	0.020
Epinephrine E_max_	Optimul	2815	−**0.31**	**0.05**	**2.21E‐10**	−**0.37**	**0.05**	**4.87E‐13**	−**0.38**	**0.06**	**1.25E‐10**	−0.18	0.07	0.017	−0.03	0.11	0.763
Ristocetin disagg	LTA	3017	0.15	0.05	1.45E‐03	**0.17**	**0.05**	**4.98E‐04**	0.14	0.06	0.013	0.17	0.07	0.017	0.08	0.10	0.438
TRAP‐6 velocity	MP	3125	**0.16**	**0.05**	**4.56E‐04**	**0.17**	**0.05**	**4.48E‐04**	0.19	0.05	7.11E‐04	0.12	0.07	0.086	0.34	0.10	1.02E‐03
U46619 Agg20	Optimul	2704	**0.25**	**0.05**	**2.50E‐07**	**0.29**	**0.05**	**1.14E‐08**	**0.26**	**0.06**	**9.04E‐06**	0.17	0.08	0.024	0.05	0.11	0.671
U46619 Agg40	Optimul	2728	**0.29**	**0.05**	**4.83E‐09**	**0.33**	**0.05**	**7.54E‐11**	**0.34**	**0.06**	**5.23E‐09**	0.14	0.07	0.071	−0.01	0.11	0.950
U46619 AUC	Optimul	2766	−**0.29**	**0.05**	**2.82E‐09**	−**0.32**	**0.05**	**1.83E‐10**	−**0.34**	**0.06**	**1.04E‐08**	−0.16	0.07	0.036	−0.05	0.11	0.663
U46619 EC_50_	Optimul	2734	**0.30**	**0.05**	**1.45E‐09**	**0.33**	**0.05**	**9.09E‐11**	**0.33**	**0.06**	**1.69E‐08**	0.16	0.07	0.030	0.02	0.11	0.827
U46619 slope	Optimul	2758	**0.25**	**0.05**	**2.54E‐07**	**0.25**	**0.05**	**1.32E‐06**	**0.24**	**0.06**	**7.35E‐05**	0.26	0.08	6.08E‐04	0.17	0.11	0.127

Significant results for platelet traits passing multiple correction, with additional results in other medication classes also shown. Associations passing multiple test correction are indicated in bold.

AA, arachidonic acid; AD, adenosine diphosphate; agg, aggregation; Agg20, effective concentration needed to reach 20% aggregation; Agg40, effective concentration needed to reach 40% aggregation; AUC, area under the concentration–aggregation curve; disagg, percent disaggregation; EC_50_, half maximal effective concentration; EC_max_, effective concentration where maximal aggregation was observed; E_max_, maximal aggregation observed; LTA, light transmission aggregometry; MP, Multiplate; NDRI, norepinephrine and dopamine reuptake inhibitor; SSRI, selective serotonin reuptake inhibitor; TRAP‐6, thrombin receptor activating peptide‐6.

To focus on specific serotonin inhibitors, we also accounted for P2Y12/ADP receptor inhibitor medication, in addition to age, sex, and aspirin use, in SSRI and SARI groups (**Table** **S7** and **Table** **S8**, respectively). SSRI associations with Optimul ADP concentration to reach 20% aggregation and LTA secondary aggregation slope to 0.98 μM were somewhat attenuated below our significance threshold, but other associations remained significant or were even strengthened (**Table** **S7**). While associations with SARI use did not pass multiple test correction, there were borderline associations that suggest SARIs may also suppress multiple platelet activation pathways. These included higher ristocetin LTA disaggregation (*P *< 0.0013), lower LTA ADP aggregation (to 0.98 μM and 1.82 μM, *P *< 0.0022 and *P *< 0.0047, respectively), lower P‐selectin percentage positive platelets in response to 20 μM ADP (*P *< 0.0052), lower WB Multiplate 3.19 cM ADP activation (*P *< 0.019), lower LTA aggregation to epinephrine (*P *< 0.022) and collagen (*P *< 0.025), and lower Optimul aggregation to TRAP‐6 (*P *< 0.0090), epinephrine (*P *< 0.027), U46619 (*P *< 0.035) and collagen (*P *< 0.050). The borderline associations of SARI with reduced ADP platelet activation were all strengthened after additional adjustment for P2Y12 inhibitor use (**Table** **S8**).

We observed fewer associations for norepinephrine‐affecting and NDRI categories. Our norepinephrine‐affecting class included tricyclic antidepressants and serotonin and norepinephrine reuptake inhibitors, which also affect serotonergic mechanisms, so it is likely that the associations observed are in part due to inhibition of the serotonin transporter. Interestingly, we found one significant NDRI association in the Multiplate platform (ADP velocity; beta = 0.351, *P *= 4.87E‐04) in a direction that indicates increased platelet reactivity. **Table** **S9** and **Table** **S10** show norepinephrine‐affecting and NDRI results, respectively.

### Depression thresholds and platelet traits

There were no statistically significant associations between CES‐D 16 or 21 in our age‐adjusted, sex‐adjusted, aspirin‐adjusted, family structure–adjusted, and AD‐adjusted models, or our models excluding AD users. Results that were nominally significant in our AD‐adjusted model were predominantly in the direction of effect opposite of that hypothesized. However, after excluding AD users, these trends were largely attenuated. Results for platelet reactivity traits and depression thresholds, including models with and without AD users, can be found in **Tables** **S11–S14**.

### Aspirin use strata and serotonin‐affecting drugs

As in our main sample analysis, we found a few significant associations within our aspirin use strata, particularly for ADP and U46619 traits in LTA and Optimul assays. The remaining, nonsignificant, associations predominantly showed trends consistent with a decrease in platelet reactivity, suggesting a synergistic antiplatelet effect for those taking aspirin and serotonin‐affecting ADs concurrently. **Table** **S15** shows results for all 26 traits analyzed in the aspirin strata.

### Boston Puerto Rican Health Study serotonin‐affecting antidepressant results

In the BPRHS main sample analysis, our findings were replicated in three out of five traits tested: U46619 effective concentration needed to reach 20% aggregation (beta = 0.322*, P *= 2.42E‐03), half maximal effective concentration (beta = 0.282, *P *= 7.85E‐03), and effective concentration needed to reach 40% aggregation (beta = 0.279, *P *= 8.71E‐03). All traits were in the same direction of effect as our FHS analysis, indicating an inhibition of platelet reactivity. Findings in the aspirin sample of BPRHS analysis were nonsignificant. Results from BPRHS regression analyses in the main sample and the aspirin subsample can be found in **Table** **S16**.

## DISCUSSION

To our knowledge, this is the largest study to date to investigate the effects of depressive symptomology and AD use, respectively, on platelet function traits derived from many different agonist pathways. We show that AD use, particularly medications affecting the serotonergic system, are strongly associated with multiple platelet aggregation traits in our main sample, and that the effect may further synergistically reduce platelet reactivity in those taking aspirin. This suggests that researchers conducting platelet function tests in nearly all contexts should not only collect and utilize information on direct antiplatelet medications, but also consider antidepressant medications with particular attention to those affecting serotonin biology.

Our results are in line with past research that demonstrated that SSRIs inhibit platelet activation and aggregation to a host of agonists, which is likely due to intraplatelet serotonin depletion.^14^, ^23^ Based on our study, these drugs seem to largely impact weaker platelet agonists such as epinephrine, ADP, and the thromboxane A2 mimetic U46619 in reaching maximal aggregation potentials normally observed. Although consistent results were observed between ADP for Optimul and LTA, the strongest results were observed overall with Optimul assays. This may be due to technical differences in the assays as previously discussed,^24^ due to the fact that LTA only tested single agonist concentrations, whereas Optimul provides a picture across concentration‐response dynamics, and that we did not test U46619 with LTA. Interestingly, U46619 is thought to depend on dense granule release, particularly of ADP, in stimulating platelet aggregation, which suggests that serotonin is also implicated, and dense granule release may be attenuated.^25^ Additionally, ADP secondary aggregation and secondary slope at low agonist concentration were significantly reduced in the context of serotonin‐affecting antidepressant drugs. This suggests a need for serotonin in secondary amplification and, given that significant positive associations were found with ADP disaggregation, less stable platelet aggregates are also being formed in individuals taking these medications.

Alternative mechanisms of serotonin‐affecting drugs’ platelet inhibitory effect have also been explored. In an *in vitro* platelet study, the SSRI citalopram was found to inhibit platelet aggregation, adhesion, and thromboxane A2 production in response to both collagen and U46619 through mechanisms other than serotonin transport blockade.^23^ In a follow‐up study, two novel mechanisms for citalopram‐induced platelet inhibition were found: inhibition of the calcium and diacylglycerol guanine nucleotide exchange factor‐1 (CalDAG‐GEFI)–mediated Rap1‐GTP formation, which facilitates fibrinogen crosslinking by turning integrin αIIb‐ßIII into its high affinity state, and competitive antagonism of the FcRϒ‐chain of the GPVI receptor.^26^ The concentration of citalopram needed to observe these effects, however, was much higher than what would be seen physiologically, leading the authors to conclude that it is unlikely to be of clinical significance. Still, it is possible that these mechanisms exert small effects in our study results. The CalDAG‐GEFI–mediated formation of GTP‐Rap1 is upregulated by a rise in cytosolic calcium ion, a shared downstream effect of many different platelet agonists. Considering that we observed traits induced by many different agonists to be negatively affected by serotonin‐affecting drugs, including strong platelet agonists such as ristocetin and collagen, it is possible that a broader mechanism, such as reduction in CalDAG‐GEFI–mediated GTP exchange, is also at play. Similarly, multiple statistically significant negative associations with collagen agonism suggests the possibility of competitive antagonism of SSRIs on GPVI. Extrapolation of these studies to our results, however, should be taken with caution as it is most likely, given the difference in strength of results, that most of the effects of serotonin‐affecting ADs on platelets is due to depletion of serotonin stores and attenuated secondary amplification of platelet activation.

While it seems clear that serotonin transporter (SERT)–acting drugs inhibit platelets, research on the clinical significance of such inhibition is much less concordant.^2^, ^3^, ^14^, ^27^, ^28^, ^29^ There are two primary implications regarding the platelet inhibitory effect of serotonin‐affecting ADs in the clinic. The first is that serotonin‐affecting ADs, most commonly SSRIs, can increase the risk of adverse bleeding. In a recent meta‐analysis of bleeding risk pertaining to SSRI use, SSRIs were associated with a 41% and 36% increase of bleeding in case‐control studies and cohort studies, respectively.^27^ This increased risk was associated with concurrent nonsteroidal anti‐inflammatory drug use and found to be mainly gastrointestinal (GI)‐related. However, an odds ratio of 1.16 was observed for intracranial hemorrhage (ICH), a serious adverse bleeding event. It is important to note that many of the studies included in this meta‐analysis were comprised of high‐risk populations, such as surgical patients, who are more prone to bleeds. Additionally, SSRIs have been implicated in higher risk for endoscopic mucosal injuries and GI duodenal perforation when used in combination with nonsteroidal anti‐inflammatory drugs, which may suggest that the increased risk of GI bleeds with SSRI treatment is not entirely antiplatelet in nature, but rather due in part to GI toxicity.^29^ Another interesting critique of past studies citing increased risk of bleeding on SSRIs, particularly in the context of ICH, is the extent to which covariates are adjusted for. In a study which adjusted for many covariates, including the concurrent diagnosis of depression, no association was found between ICH and SSRI use, suggesting that depression’s link to unfavorable lifestyle factors such as lack of adherence to medical treatment, lack of exercise, poor diet, or increased risk of alcohol and drug use may have confounded past positive association results with ICH in SSRI users.^28^

In addition to bleeding risk, the antiplatelet effects of serotonin‐affecting ADs have driven the theory that these drugs may be beneficial to cardiovascular health through a decrease in thrombotic risk. The literature in this realm to date, however, has been inconsistent. While some studies have found SSRIs to decrease the risk of myocardial infarction and overall cardiovascular mortality, others have found no significant differences, even increased risk of thrombotic events,^29^, ^30^ or mixed risk with protection against stroke but not myocardial infarction (MI).^31^ Differences in study design (case/control, event definition) and risk factor stratification may account for some of the discrepancies. Once again, depression as an affective disorder adds complexity to past results, as the improvement of mood by SSRIs, or underlying concurrent depression, may be driving cardiovascular associations through lifestyle factors instead of antiplatelet effects. Recently, a large study of 24,155 MI cases and 120,776 controls in a primary healthcare database found significant protective effects against MI in users of SSRIs (adjusted odds ratio 0.86), and reported the first association of trazodone (SARI) being protective for MI (adjusted odds ratio 0.76).^32^ This study suggests that at typical low doses of trazodone, where serotonin transport is less inhibited, there could be direct antithrombotic effects via antagonism of the 5‐HT2A receptor, which is known to be expressed and functional on the platelet membrane.^33^ In our study, we found a suggestive pattern of association of SARI use (*n *= 50), all in the direction of platelet reactivity inhibition, with similar or stronger effect sizes observed as with SSRIs (*n *= 362). This suggests with a larger sample of individuals taking SARIs the associations likely would have reached stringent significance and supports the possibility of multiple distinct influences of serotonin‐influencing medications on platelet reactivity that may ultimately contribute to bleeding and thrombotic risks.

The results of our study could lend support to either hypothesis of clinical relevance. The role of platelets in arterial thrombosis is well established, and platelets have long been a proven drug target for secondary CVD prevention and management. Thus, it follows that the antiplatelet effects of serotonin‐affecting ADs could lead to improvements in cardiovascular health. Given that CVD and depression are often comorbid, adding a serotonin‐affecting AD to the regimen of those at high risk for thrombosis may bear parallels to dual antiplatelet therapy of aspirin and an ADP inhibitor, which has been found to decrease the risk of secondary cardiovascular events when compared with aspirin alone.^34^ Conversely, it is intuitive that inhibiting platelets too much could eventually lead to adverse bleeding. Use of aspirin in primary prevention has grown out of favor in recent years. Still, whether aspirin or other antiplatelet therapy combinations are applied in primary or secondary prevention, our results suggest there could be synergistic pharmacodynamic effects of serotonin‐affecting ADs with these medications that might be beneficial in patients at high risk for thrombosis, while also potentially increasing the likelihood of bleeding outcomes, a trade‐off that might not be overall favorable in low‐risk individuals. While it is unlikely that SSRIs significantly increase the risk of bleeding in the general population,^27^ those at a higher risk for bleeding may benefit more from a nonserotonergic AD such as the NDRI bupropion, which has been shown to be well tolerated and which seemed to exert minimal effects on platelets in our study.^35^ Overall, future large‐scale, comprehensively controlled studies of serotonin‐affecting AD use and both bleeding and cardiovascular event risk, respectively, are needed to fully elucidate the clinical significance of serotonin‐affecting AD’s antiplatelet effects.

Our results for depressive symptomology and platelet reactivity were nonsignificant when adjusting for antidepressant use. It has been postulated that 5HT2A platelet expression and serotonin agonism is higher in depression.^14^ Platelets express glutamate receptors, and glutamatergic dysfunction in depression may also affect platelet reactivity.^36^, ^37^ Our study did not test serotonin or glutamate as agonists, and may, thus, have missed some direct links. Still, with otherwise fairly comprehensive platelet reactivity phenotyping and largely null findings, it seems unlikely that platelet hyperreactivity is a large factor in depressive comorbidity.

It is important to note that our study comes with limitations. First, although we tested many platelet variables and pathways, it should be acknowledged that in any platelet study assay choices (e.g., agonist concentrations) and preanalytic variables (e.g., tube anticoagulant choice, agonist batch effects, or centrifugation settings) can influence results. In our study we took steps to reduce such effects like dedicated agonist batches across the study, standardized methods, and using few lab personnel. Second, our analysis of AD use is collected cross‐sectionally, and thus, we were unable to direct or account for dose or duration of therapy. Interestingly, however, past research indicates that therapeutic doses of SSRIs maximally inhibit the serotonin transporter and fully exert antiplatelet effects, so this may not be a large confounding factor.^29^, ^38^ Third, we define AD use via self‐report and cannot rule out misreport or noncompliance with medication. Given the nature of depression, and because antidepressants are often prescribed for long periods of time, it is possible that those who report taking ADs are not fully adhering to treatment, which could confound our results. However, given that 99.9% of the Framingham participants with platelet phenotyping physically brought their medication bottles to their exam, it is likely we had excellent coverage of prescriptions, unless some individuals chose not to report AD out of a sense stigmatism. Even if there were such effects, or medication noncompliance, we would expect that would bias toward our null hypothesis and weaken any results. Although our primary analysis population was limited primarily to those of European ancestry, we were able to replicate several of our U46619 Optimul significant associations, despite being restricted by statistical power, in an independent, non‐European cohort (BPRHS), suggesting that our findings may generalize to additional populations. Findings for U46619 and serotonin‐affecting medication within the aspirin strata in BPRHS did not replicate, although the effect direction trend was similar, indicating decreased platelet activation. This may be due to limited power with *n *= 62 reporting being on aspirin and serotonin‐affecting medications. Reliance on self‐reported aspirin, rather than the sensitive LTA‐AA assay used in FHS, may have also played a role and resulted in some substratum misassignment in the BPRHS replication and bias toward the null hypothesis. Finally, our depression and severe depression variables were derived using the CES‐D survey rather than a clinical diagnosis in medical record, which limits our ability to draw firm conclusions about platelet hyperreactivity and depression such as would be obtained with validated *Diagnostic* *and Statistical Manual of Mental Disorders* (Fifth Edition) criteria being met.^1^

Conversely, our study presents many strengths in the field of platelet function testing. We analyzed platelet activation and reactivity traits spanning multiple assays, agonists, and in the context of both whole blood and platelet‐rich plasma, which allows insight into the antiplatelet mechanisms at play in serotonin‐affecting AD therapy. Additionally, we present a large sample size consisting of predominantly healthy middle‐aged individuals, which may be well generalizable to the overall population. While our study results show clear antiplatelet effects of serotonin‐affecting ADs, the average age of participants is still somewhat low to analyze clinical associations such as bleeding or cardiovascular events; thus, future examinations of the Generation 3 and NOS cohorts of the FHS research on serotonin‐affecting AD use and risk of bleeding and cardiovascular event, respectively, may allow better understanding of the clinical significance of serotonin‐affecting AD antiplatelet effects and may ultimately support clinical platelet function testing in prescribing and monitoring ADs.

## FUNDING

This research was primarily supported by a special Population Sciences funding award to A.D.J. from the National Heart, Lung, and Blood Institute (NHBLI) Intramural Research program. The Framingham Heart Study (FHS) acknowledges the support of Contracts NO1‐HC‐25195, HHSN268201500001I, and 75N92019D00031 from the NHLBI and grants HL107385, HL126136, HL93328, HL142983, HL143227, and HL131532 for this research. P.J.C.A., M.V.C. and T.D.W were also supported by The British Heart Foundation (RG/19/8/34500). C.O., K.L.T., and M.G. and the BPRHS were supported by NHLBI P50 HL105185, and National Institute of Aging P01 AG023394 and R01 AG055948.

## CONFLICT OF INTEREST

The authors declared no competing interests for this work.

## AUTHOR CONTRIBUTIONS

A.D.J., B.B.N, F.T., J.G., K.L.T., M‐H.C., M.V.C., and T.D.W. wrote the manuscript. A.D.J., A.L., C.O., C.W.M., K.L.T., M.G., M‐H.C., M.V.C., P.C.J.A., T.D.W., and Z.S. designed the research. A.D.J., A.L., C.O., C.W.M., and Z.S. performed the research. A.D.J., F.T., J.G., M‐H.C., and M.V.C. analyzed the data. M.V.C., P.C.J.A., and T.D.W. contributed new reagents/analytical tools.

## DISCLAIMER

The views expressed in this manuscript are those of the authors and do not necessarily represent the views of the National Heart, Lung, and Blood Institute; the National Institutes of Health; or the US Department of Health and Human Services.

## Supporting information

Supplementary Material

Fig S1

Supplementary Material

## References

[1] Williams, M.S. Platelets and depression in cardiovascular disease: a brief review of the current literature. World J. Psychiatry 2, 114–123 (2012). 24175177 10.5498/wjp.v2.i6.114 PMC3782186

[2] Vieweg, W.V.R. *et al*. Treatment of depression in patients with coronary heart disease. Am. J. Med. 119, 567–573 (2006). 16828625 10.1016/j.amjmed.2006.02.037

[3] Yekehtaz, H. , Farokhnia, M. & Akhondzadeh, S. Cardiovascular considerations in antidepressant therapy: an evidence‐based review. J. Tehran Heart Cent. 8, 169–176 (2013). 26005484 PMC4434967

[4] Musselman, D.L. *et al*. Platelet activation and secretion in patients with major depression, thoracic aortic atherosclerosis, or renal dialysis treatment. Depress Anxiety 15, 91–101 (2002). 12001177 10.1002/da.10020

[5] Markovitz, J.H. , Shuster, J.L., Chitwood, W.S., May, R.S. & Tolbert, L.C. Platelet activation in depression and effects of sertraline treatment: an open‐label study. Am. J. Psychiatry 157, 1006–1008 (2000). 10831484 10.1176/appi.ajp.157.6.1006

[6] Laghrissi‐Thode, F. , Wagner, W.R., Pollock, B.G., Johnson, P.C. & Finkel, M.S. Elevated platelet factor 4 and beta‐thromboglobulin plasma levels in depressed patients with ischemic heart disease. Biol. Psychiatry 42, 290–295 (1997). 9270907 10.1016/S0006-3223(96)00345-9

[7] Pollock, B.G. , Laghrissi‐Thode, F. & Wagner, W.R. Evaluation of platelet activation in depressed patients with ischemic heart disease after paroxetine or nortriptyline treatment. J. Clin. Psychopharmacol. 20, 137–140 (2000). 10770450 10.1097/00004714-200004000-00004

[8] Lederbogen, F. *et al*. Increased platelet aggregability in major depression? Psychiatry Res. 102, 255–261 (2001). 11440776 10.1016/s0165-1781(01)00259-1

[9] Shimbo, D. *et al*. Exaggerated serotonin‐mediated platelet reactivity as a possible link in depression and acute coronary syndromes. Am. J. Cardiol. 89, 331–333 (2002). 11809437 10.1016/s0002-9149(01)02236-6

[10] Walsh, M.T. , Dinan, T.G., Condren, R.M., Ryan, M. & Kenny, D. Depression is associated with an increase in the expression of the platelet adhesion receptor glycoprotein Ib. Life Sci. 70, 3155–3165 (2002). 12008098 10.1016/s0024-3205(02)01569-2

[11] Gomez‐Gil, E. *et al*. Platelet 5‐HT2A‐receptor‐mediated induction of aggregation is not altered in major depression. Hum. Psychopharmacol. 17, 419–424 (2002). 12457378 10.1002/hup.429

[12] Parakh, K. , Sakhuja, A., Bhat, U. & Ziegelstein, R.C. Platelet function in patients with depression. South Med. J. 101, 612–617 (2008). 18475223 10.1097/SMJ.0b013e318172f732

[13] Musselman, D.L. *et al*. Exaggerated platelet reactivity in major depression. Am. J. Psychiatry 153, 1313–1317 (1996). 8831440 10.1176/ajp.153.10.1313

[14] Halperin, D. & Reber, G. Influence of antidepressants on hemostasis. Dialogues Clin. Neurosci. 9, 47–59 (2007). 17506225 10.31887/DCNS.2007.9.1/dhalperin PMC3181838

[15] Dietrich‐Muszalska, A. & Wachowicz, B. Platelet haemostatic function in psychiatric disorders: effects of antidepressants and antipsychotic drugs. World J. Biol. Psychiatry 18, 564–574 (2017). 27112326 10.3109/15622975.2016.1155748

[16] Maurer‐Spurej, E. , Pittendreigh, C. & Solomons, K. The influence of selective serotonin reuptake inhibitors on human platelet serotonin. Thromb. Haemost. 91, 119–128 (2004). 14691577 10.1160/TH03-05-0330

[17] Splansky, G.L. *et al*. The third generation cohort of the national heart, lung, and blood institute's Framingham heart study: design, recruitment, and initial examination. Am. J. Epidemiol. 165, 1328–1335 (2007). 17372189 10.1093/aje/kwm021

[18] Andersson, C. , Johnson, A.D., Benjamin, E.J., Levy, D. & Vasan, R.S. 70‐year legacy of the Framingham Heart Study. Nat. Rev. Cardiol. 16, 687–698 (2019). 31065045 10.1038/s41569-019-0202-5

[19] Tucker, K.L. *et al*. The Boston Puerto Rican Health Study, a longitudinal cohort study on health disparities in Puerto Rican adults: challenges and opportunities. BMC Public Health 10, 107 (2010). 20193082 10.1186/1471-2458-10-107 PMC2848197

[20] Cosco, T.D. , Prina, M. , Stubbs, B. & Wu, Y.‐T. Reliability and validity of the center for epidemiologic studies depression scale in a population‐based cohort of middle‐aged U.S. adults. J. Nurs. Meas. 25, 476–485 (2017). 29268830 10.1891/1061-3749.25.3.476

[21] Moscicki, E. , Rae, D.S. , Reiger, E. & Locke, B. Depression among Mexican Americans, Cubans and Puerto Ricans. In Health and behavior: Research agenda for Hispanics (Simon Bolivar Research Monograph Series I) (eds. Gaviria, M. and Arana, J. ) 145–159 (University of Illinois Press, Chicago, IL, 1987).

[22] Harrison, P. , Bethel, M.A., Kennedy, I., Dinsdale, R., Coleman, R. & Holman, R.R. Comparison of nine platelet function tests used to determine responses to different aspirin dosages in people with type 2 diabetes. Platelets 30, 521–529 (2019). 29985735 10.1080/09537104.2018.1478402

[23] Roweth, H.G. *et al*. Citalopram inhibits platelet function independently of SERT‐mediated 5‐HT transport. Sci. Rep. 8, 3494 (2018). 29472624 10.1038/s41598-018-21348-3 PMC5823918

[24] Chan, M.V. , Leadbeater, P.D. , Watson, S.P. & Warner, T.D. Not all light transmission aggregation assays are created equal: qualitative differences between light transmission and 96‐well plate aggregometry. Platelets 29, 686–689 (2018). 29715047 10.1080/09537104.2018.1466388 PMC6178086

[25] Armstrong, P.C. *et al*. In the presence of strong P2Y12 receptor blockade, aspirin provides little additional inhibition of platelet aggregation. J. Thromb. Haemost. 9, 552–561 (2011). 21143373 10.1111/j.1538-7836.2010.04160.x PMC3064407

[26] Roweth, H.G. *et al*. Two novel, putative mechanisms of action for citalopram‐induced platelet inhibition. Sci. Rep. 8, 16677 (2018). 30420683 10.1038/s41598-018-34389-5 PMC6232110

[27] Laporte, S. *et al*. Bleeding risk under selective serotonin reuptake inhibitor (SSRI) antidepressants: a meta‐analysis of observational studies. Pharmacol. Res. 118, 19–32 (2017). 27521835 10.1016/j.phrs.2016.08.017

[28] Liu, L. *et al*. Selective serotonin reuptake inhibitors and intracerebral hemorrhage risk and outcome. Stroke 51, 1135–1141 (2020). 32126942 10.1161/STROKEAHA.119.028406 PMC7147963

[29] de Abajo, F.J. Effects of selective serotonin reuptake inhibitors on platelet function: mechanisms, clinical outcomes and implications for use in elderly patients. Drugs Aging 28, 345–367 (2011). 21542658 10.2165/11589340-000000000-00000

[30] Chen, Y. , Guo, J.J., Hong, L., Wulsin, L. & Patel, N.C. Risk of cerebrovascular events associated with antidepressant use in patients with depression: a population‐based, nested case‐control study. Ann. Pharmacother. 42, 177–184 (2008). 18212255 10.1345/aph.1K369

[31] Douros, A. , Dell’Aniello, S., Dehghan, G., Boivin, J.F. & Renoux, C. Degree of serotonin reuptake inhibition of antidepressants and ischemic risk: a cohort study. Neurology 93, e1010–e1020 (2019). 31391245 10.1212/WNL.0000000000008060 PMC6745737

[32] Alqdwah‐Fattouh, R. *et al*. Differential effects of antidepressant subgroups on risk of acute myocardial infarction: a nested case‐control study. Br. J. Clin. Pharmacol. 86, 2040–2050 (2020). 32250461 10.1111/bcp.14299 PMC7495291

[33] Lin, O.A. , Karim, Z.A., Vemana, H.P., Espinosa, E.V.P. & Khasawneh, F.T. The antidepressants 5‐HT2A receptor antagonists pizotifen and cyproheptadine inhibit serotonin‐enhanced platelet function. PLoS One 9, e87026 (2014). 24466319 10.1371/journal.pone.0087026 PMC3900701

[34] Udell, J.A. *et al*. Long‐term dual antiplatelet therapy for secondary prevention of cardiovascular events in the subgroup of patients with previous myocardial infarction: a collaborative meta‐analysis of randomized trials. Eur. Heart J. 37, 390–399 (2016). 26324537 10.1093/eurheartj/ehv443

[35] Fava, M. *et al*. 15 years of clinical experience with bupropion HCl: from bupropion to bupropion SR to bupropion XL. Prim. Care Companion J. Clin. Psychiatry 7, 106–113 (2005). 16027765 10.4088/pcc.v07n0305 PMC1163271

[36] Chen, H. Possible role of platelet GluR1 receptors in comorbid depression and cardiovascular disease. Cardiovasc. Psychiatry Neurol. 2009, 424728 (2009). 20029621 10.1155/2009/424728 PMC2790151

[37] Pereira, V.S. & Hiroaki‐Sato, V.A. A brief history of antidepressant drug development: from tricyclics to beyond ketamine. Acta Neuropsychiatr. 30, 307–322 (2018). 29388517 10.1017/neu.2017.39

[38] Abdelmalik, N. *et al*. Effect of the selective serotonin reuptake inhibitor paroxetine on platelet function is modified by a SLC6A4 serotonin transporter polymorphism. J. Thromb. Haemost. 6, 2168–2174 (2008). 18983505 10.1111/j.1538-7836.2008.03196.x

