# Supplementary material for: Serotonin‐Affecting Antidepressant Use in Relation to Platelet Reactivity
Source: Clin Pharmacol Ther. 2022 Jan 10;111(4):909–18. doi: 10.1002/cpt.2517 (PMC9305794; doi:10.1002/cpt.2517)
Supplement: Supplementary file 1 — Supplementary Material [file CPT-111-909-s001.docx]

**Supplemental Text to Grech *et al.***

*Blood draw.* Participant blood was collected following an overnight fast via venipuncture of the antecubital vein in both cohorts. For the Framingham study, participants were in a supine position in the research center, and phlebotomists used a 21 gauge safety lok blood collection set (BD Biosciences; San Jose, CA). For the BPRHS, blood was drawn in a sitting position in the participant’s home into 4.5 mL sodium citrate (3.2%) tubes (BD; San Jose, CA). Samples were taken to the University of Massachusetts Lowell within two hours. All assays were conducted within three hours of participants’ blood draw.

In Framingham we collected one Hirudin blood tube (3.0mL; Diapharma; West Chester, OH) for whole blood (WB)-based platelet assays: Flow cytometry (ADP-stimulated), Total-Thrombus Formation Analysis System Plus (T-TAS), and Multiplate impedance aggregometry (MP). Hirudin tubes incubated at room temperature for at least 30 minutes prior to use.

In Framingham three 4.5 mL sodium citrate (3.2%) tubes (BD; San Jose, CA) were collected for platelet rich plasma (PRP)-based assays: Flow cytometry (ADP stimulated), light transmission aggregometry (LTA), and a custom 96-well plate light absorbance-based assay (Optimul).

*Isolation of PRP.* We centrifuged sodium citrate tubes at 200 x g for 10 minutes (Sorvall ST8 centrifuge, Thermo Sci 75003181 rotor) to isolate PRP, and 1500 x g for 15 minutes to isolate platelet poor plasma (PPP). Sodium citrate tubes incubated at room temperature for at least 15 minutes prior to any centrifugation.

*Platelet agonist sources.* A vast majority of our platelet assay agonists were delivered in large lots from Bio/Data Corporation (Horsham, PA). Exceptions to this were MP AA/Aspi test (Roche; Basel, Switzerland), U46619 (Cayman Biochemical, Ann Arbor, MI, USA), and a small number of Optimul plates made in London, UK with agonists from: Sigma-Aldrich (AA; Poole, UK), Labmedics (ADP, epinephrine; Salford, Manchester, UK), Takeda (collagen; London, UK), Helena Biosciences (ristocetin; Gateshead, Tyne & Wear, UK), Bachem (TRAP-6; St. Helens, Merseyside, UK), and Enzo (U46619; Exeter, UK).

*Platelet reactivity traits.* Our study contained many distinct platelet reactivity assays spanning WB and PRP milieus and multiple agonists at varying concentrations, totaling to 130 platelet reactivity traits. Due to the breadth of assays used in this study, many platelet traits were available to analyze.

*Whole blood impedance aggregometry.* We utilized a 5-channel Multiplate Analyzer (Roche Diagnostics; Manheim, Germany) for collection of WB impedance aggregometry traits. Aggregation, AUC, and velocity for each agonist were reported as platelet traits. 300 μL of saline and WB, respectively, were added to each of the five channels and incubated at 37C for three minutes. We then added ADP (final concentration: 3.19 μM), collagen (final concentration: 0.061 mg/mL), Thrombin Receptor Activating Peptide-6 (TRAP-6) (final concentration: 4.48 μM), and AA/ASPI test (final concentration 0.5 mM), to channels one through five, respectively. A Correlation Coefficient value greater than 0.985 and a Diff value below 20 were required for inclusion of data points in our analysis.

*Shear-stress mediated platelet thrombus formation*. We utilized the “PL chip” and “mid-shear” 1500 1/s settings of the Total-Thrombus Formation Analysis System (T-TAS) Plus (Zacros; Tokyo, Japan) for assessment of shear stress mediated platelet occlusion. T-TAS AUC, occlusion speed, occlusion start, and occlusion time were analyzed as platelet traits. 320 μL of participant whole blood was added to T-TAS PL-specific reservoirs, secured with an overcap, and placed onto the well of a PL chip prior to test initiation. The assay was automatically ended after a total pressure exceeded base kPa + 60 (occlusion threshold) or ten minutes had passed.

*Light Transmission Aggregometry*. We collected PRP-based platelet reactivity traits with an eight channel PAP-8E light transmission aggregometer (Bio/Data; Horsham, PA) in accordance with International Society on Thrombosis and Hemostasis guidelines [1]. LTA AUC, percent final aggregation, percent maximal aggregation, primary aggregation, and primary slope were collected for each agonist. Additionally, we collected traits for lag time to AA and collagen, secondary aggregation and secondary slope for ADP and epinephrine, and disaggregation to ADP and ristocetin. 25 μL of 0.9% normal saline and 225 μL of participant PPP in an assay-specific cuvette used to “blank” each test cell, and eight cuvettes were loaded with 225 μL of participant PRP to incubate for two minutes at 37C with a magnetic stir bar. AA (final concentration: 1.6 mM), three different concentrations of ADP (0.95 μM, 1.82 μM, and 5.71 μM), collagen (190 μg/mL), epinephrine (100 μM), TRAP-6 (15.0 μM), and Agg-Recetin (1.5 mg/mL) were added to channels one through eight, respectively.

*Optimul 96-well plate assay*. The Optimul 96-well plate assay was used to assess platelet reactivity to varying concentrations of seven different platelet agonists [2]. Plates required for the assay were primarily made in-house in Framingham, MA, USA, with the exception of a small proportion made in the UK. The protocol for doing so can be found below. Optimul AUC mean, EC50, EMax, concentration to reach 20% (Agg20) and 40% aggregation (Agg40), ECMax, and slope were collected as traits for each of the following agonists: AA, ADP, collagen, epinephrine, ristocetin, TRAP-6, and U46619. Plates required for the assay were pre-made by gel coating with a buffer composed of monobasic NaH2PO4 monohydrous, dibasic Na2HPO4 anhydrous, DI water, gelatin, and Tween-20. After the plates dried, AA (0.03, 0.06, 0.11, 0.19, 0.33, 0.57 and 1.0 mM), ADP (0.005, 0.02, 0.10, 0.44, 1.98, 8.89 and 40 µM), collagen (0.01, 0.04, 0.16, 0.62, 2.5, 10 and 40 µg/ml), epinephrine (0.0004, 0.001, 0.01, 0.06, 0.33, 1.82 and 10 µM), ristocetin (0.14, 0.24, 0.43, 0.75, 1.31, 2.29 and 4 mg/ml), TRAP-6 (0.03, 0.11, 0.36, 1.17, 3.79, 12.3 and 40 µM), and U46619 (0.005, 0.02, 0.10, 0.44, 1.98, 8.89 and 40 µM) were added from highest to lowest concentration and plates were frozen at -80C and lyophilized for at least eight hours.

To conduct the assay, PPP and PRP were added to relevant wells for controls and 40 μL of PRP was added to wells A-H in each well containing agonist. Plates were read at 595 nm (ELx800, BioTek, Winooski, VT, USA) before and after a five minute shake at 1200 RPM and 37C. Concentration response curves were constructed using nplr R package (<https://cran.r-project.org/web/packages/nplr/index.html>.

*Flow cytometry to measure ADP-driven platelet activation*. Flow cytometry data was acquired using the BD Accuri C6 instrument, and the BD Accuri analysis software (BD Biosciences; San Jose, CA, USA) was used for gating and export of data for analysis by linear mixed effects models in R. Flow cytometry traits, all of which measured in both PRP and WB, include percent positive PAC-1 and P-selectin platelets; mean fluorescence intensity differential for PAC-1 and P-selectin, and normal saline estimated platelet count. We prepared a 1:40 dilution of both PRP and WB by adding 10 μL of PRP and WB, respectively, to 390 μL of phosphate buffered solution. 45 μL of each dilution mix was added to 5 μL of normal saline and 5 μL of 200 μM ADP, respectively, and incubated at 37C for 15 minutes. Following incubation, 50 μL of antibody cocktail containing 1:20 anti-CD61 antibody (PerCP-Cy5.5 Mouse Anti-Human CD61 Clone VI-PL2), 1:5 anti-CD62P antibody (APC Mouse Anti-Human CD62P; Clone AK-4), and 1:5 anti-PAC-1 antibody (FITC Mouse Anti-Human PAC-1; Clone PAC-1) was added to each tube and incubated at 37C for 15 minutes. Finally, 1150 μL of staining mix containing three parts stain buffer and one part cytofix solution (BD Biosciences; San Jose, CA, USA) was added to complete the sample preparations.

Each sample was run on a “slow” flow-rate and a primary stop condition set at “10,000 CD61+ events.” Gates were drawn based on experiments with different antibody combination and stimulated or unstimulated conditions, and were cross-checked across random samples throughout every month of the exam period and fine-tuned before developing a final analysis template.

*Derivation of multiple testing correction threshold based on principal components analyses for independent traits.* To account for multiple testing, we used principal components analysis (PCA) to derive the effective number of independent traits that retained 90% of total trait variation and used the number to compute the Bonferroni correction threshold [3,4]. As each may have a different sample size, we applied PCA to three samples, 1) the first sample had all 130 platelet traits (n=543), 2) the second included the 126 traits that had a sample size of at least 2,000 (excluding 4 Total-Thrombus Formation Analysis traits), and 3) the third included the 121 traits that had a sample size of at least 2,500 (further excluding 3 thrombin activating peptide-6 and 2 arachidonic acid traits). PCA indicated that 40, 41 and 41 principal components were required to retain 90% total trait variation in the first, second, and third sample, respectively. To accommodate the loss of some samples and traits, we selected a more conservative number of 45 to be our effective number of independent traits. We defined the Bonferroni correction threshold as 0.05/(45*2) = 5.55E-4 to further account for the separate approaches of analyzing depression and antidepressant variables in our primary analyses.

Supplemental Text references

1. Cattaneo, M., et al., Recommendations for the Standardization of Light Transmission Aggregometry: A Consensus of the Working Party from the Platelet Physiology Subcommittee of SSC/ISTH. J Thromb Haemost, 2013.

2. Chan, M.V., et al., Optical multichannel (optimul) platelet aggregometry in 96-well plates as an additional method of platelet reactivity testing. Platelets, 2011. 22(7): p. 485-94.

3. Gao, X., Starmer, J., Martin, E.R. A multiple testing correction method for genetic association studies using correlated single nucleotide polymorphisms. Genet Epidemiol, 2008 32(4): p. 361-9.

4. Mardia, K.T., Kent, J.T., Bibby, J.M. Multivariate analysis. London: Academic Press, 1979.

**PERL programming utilized to assign (no=0, yes=1) values to BPRHS participants based on their self-reported medication text strings. Similar code was utilized in FHS with slight differences to match the text strings within the medication bag file that encoded ATCC standard drugs.**

#/usr/local/bin/perl

use strict;

open(IN1,"<8yr_main_06102021.txt") || die "cant find1";

my $line = <IN1>;

chomp($line);

my (%adepr,%ssri,%sari,%saaff,%noraff,%sandnaff,%tca,%ndri,%snri,%citalo,%sertral,%escitalo,%fluoxe,%paroxet);

my (%vilazo,%fluvoxa,%trazodone,%nefazod,%vortio,%mirtaz,%bupropion,%desipramine,%amitriptyline,%doxepin);

my (%nortriptyl,%imipram,%protriptyl,%duloxet,%venlafax,%desvenlaf);

my %all;

#PRINT HEADING LINE

print "Id\tAntidepressants\tSerotoninAffecting\tSSRI\t\Citalopram\tSertraline\tEscitalopram\tFluoxetine\t";

print "Paroxetine\tVilazodone\tFluvoxamine\tSARI\tTrazodone\tNefazodone\tVortioxetine\tTCA\tMirtazapine\t";

print "NeuroepiAffecting\tNDRI\tBupropion\tDesipramine\tSertAndNorAff\tAmitriptyline\tDoxepin\tNortriptyline\t";

print "Imipramine\tProtriptyline\tSNRI\tDuloxetin\tVenlafaxine\tDesvenlafaxine\n";

#READ THROUGH FILE AND MATCH DRUGS

while ($line = <IN1>)

{

chomp($line);

my @a = split(/\t/,$line);

my $id = $a[1]; # BPRHS ID

$all{$id}=$line; # store BPRHS data

# antidepressants

if($line =~ /.*[Ee]scitalopram.*/) { $adepr{$id}++; $ssri{$id}++; $saaff{$id}++; $escitalo{$id}++; }

elsif($line =~ /.*[Cc]italopram.*/) { $adepr{$id}++; $ssri{$id}++; $saaff{$id}++; $citalo{$id}++; }

if($line =~ /.*[Cc]elexa.*/) { $adepr{$id}++; $ssri{$id}++; $saaff{$id}++; $citalo{$id}++; }

if($line =~ /.*[Ss]ertraline.*/) { $adepr{$id}++; $ssri{$id}++; $saaff{$id}++; $sertral{$id}++; }

if($line =~ /.*[Ff]luoxetine.*/) { $adepr{$id}++; $ssri{$id}++; $saaff{$id}++; $fluoxe{$id}++; }

if($line =~ /.*[Pp]aroxetine.*/) { $adepr{$id}++; $ssri{$id}++; $saaff{$id}++; $paroxet{$id}++; }

if($line =~ /.*[Vv]ilazodone.*/) { $adepr{$id}++; $ssri{$id}++; $saaff{$id}++; $vilazo{$id}++; }

if($line =~ /.*[Ff]luvoxamine.*/) { $adepr{$id}++; $ssri{$id}++; $saaff{$id}++; $fluvoxa{$id}++; }

if($line =~ /.*[Tt]razodone.*/) { $adepr{$id}++; $sari{$id}++; $saaff{$id}++; $trazodone{$id}++; }

if($line =~ /.*[Nn]efazodone.*/) { $adepr{$id}++; $sari{$id}++; $saaff{$id}++; $nefazod{$id}++; }

if($line =~ /.*[Vv]ortioxetine.*/) { $adepr{$id}++; $saaff{$id}++; $vortio{$id}++; }

if($line =~ /.*[Mm]irtazapine.*/) { $adepr{$id}++; $tca{$id}++; $saaff{$id}++; $mirtaz{$id}++; }

if($line =~ /.*[Bb]upropion.*/) { $adepr{$id}++; $ndri{$id}++; $noraff{$id}++; $bupropion{$id}++; }

if($line =~ /.*[Ww]ellbutrin.*/) { $adepr{$id}++; $ndri{$id}++; $noraff{$id}++; $bupropion{$id}++; }

if($line =~ /.*[Dd]esipramine.*/) { $adepr{$id}++; $tca{$id}++; $noraff{$id}++; $desipramine{$id}++; }

if($line =~ /.*[Aa]mitriptyline.*/) { $adepr{$id}++; $tca{$id}++; $saaff{$id}++; $noraff{$id}++; $sandnaff{$id}++; $amitriptyline{$id}++; }

if($line =~ /.*[Dd]oxepin.*/) { $adepr{$id}++; $tca{$id}++; $saaff{$id}++; $noraff{$id}++; $sandnaff{$id}++; $doxepin{$id}++; }

if($line =~ /.*[Nn]ortriptyline.*/) { $adepr{$id}++; $tca{$id}++; $saaff{$id}++; $noraff{$id}++; $sandnaff{$id}++; $nortriptyl{$id}++; }

if($line =~ /.*[Ii]mipramine.*/) { $adepr{$id}++; $tca{$id}++; $saaff{$id}++; $noraff{$id}++; $sandnaff{$id}++; $imipram{$id}++; }

if($line =~ /.*[Pp]rotryptyline.*/) { $adepr{$id}++; $tca{$id}++; $saaff{$id}++; $noraff{$id}++; $sandnaff{$id}++; $protriptyl{$id}++; }

if($line =~ /.*[Dd]uloxetin.*/) { $adepr{$id}++; $snri{$id}++; $saaff{$id}++; $noraff{$id}++; $sandnaff{$id}++; $duloxet{$id}++; }

if($line =~ /.*[Dd]esvenlafaxine.*/) { $adepr{$id}++; $snri{$id}++; $saaff{$id}++; $noraff{$id}++; $sandnaff{$id}++; $desvenlaf{$id}++; }

elsif($line =~ /.*[Vv]enlafaxine.*/) { $adepr{$id}++; $snri{$id}++; $saaff{$id}++; $noraff{$id}++; $sandnaff{$id}++; $venlafax{$id}++; }

}

close(IN1);

#PRINT OUT DATA PLUS NEW ANTIDEPRESSANT INDICATOR VARIABLES

for (keys %all)

{

my $id = $_;

my $iddata = $all{$_};

print "$id\t";

# Antidepressants

if($adepr{$_}) { print "1\t"; } else { print "0\t"; }

if($saaff{$_}) { print "1\t"; } else { print "0\t"; }

if($ssri{$_}) { print "1\t"; } else { print "0\t"; }

if($citalo{$_}) { print "1\t"; } else { print "0\t"; }

if($sertral{$_}) { print "1\t"; } else { print "0\t"; }

if($escitalo{$_}) { print "1\t"; } else { print "0\t"; }

if($fluoxe{$_}) { print "1\t"; } else { print "0\t"; }

if($paroxet{$_}) { print "1\t"; } else { print "0\t"; }

if($vilazo{$_}) { print "1\t"; } else { print "0\t"; }

if($fluvoxa{$_}) { print "1\t"; } else { print "0\t"; }

if($sari{$_}) { print "1\t"; } else { print "0\t"; }

if($trazodone{$_}) { print "1\t"; } else { print "0\t"; }

if($nefazod{$_}) { print "1\t"; } else { print "0\t"; }

if($vortio{$_}) { print "1\t"; } else { print "0\t"; }

if($tca{$_}) { print "1\t"; } else { print "0\t"; }

if($mirtaz{$_}) { print "1\t"; } else { print "0\t"; }

if($noraff{$_}) { print "1\t"; } else { print "0\t"; }

if($ndri{$_}) { print "1\t"; } else { print "0\t"; }

if($bupropion{$_}) { print "1\t"; } else { print "0\t"; }

if($desipramine{$_}) { print "1\t"; } else { print "0\t"; }

if($sandnaff{$_}) { print "1\t"; } else { print "0\t"; }

if($amitriptyline{$_}) { print "1\t"; } else { print "0\t"; }

if($doxepin{$_}) { print "1\t"; } else { print "0\t"; }

if($nortriptyl{$_}) { print "1\t"; } else { print "0\t"; }

if($imipram{$_}) { print "1\t"; } else { print "0\t"; }

if($protriptyl{$_}) { print "1\t"; } else { print "0\t"; }

if($snri{$_}) { print "1\t"; } else { print "0\t"; }

if($duloxet{$_}) { print "1\t"; } else { print "0\t"; }

if($venlafax{$_}) { print "1\t"; } else { print "0\t"; }

if($desvenlaf{$_}) { print "1\t"; } else { print "0\t"; }

print "$iddata\n";

}
