# Supplementary material for: Serotonin‐Affecting Antidepressant Use in Relation to Platelet Reactivity
Source: Clin Pharmacol Ther. 2022 Jan 10;111(4):909–18. doi: 10.1002/cpt.2517 (PMC9305794; doi:10.1002/cpt.2517)

Figure S1. Final percent aggregation to arachidonic acid (AA; 1.6mM) in platelet rich plasma (PRP) normalized to platelet poor plasma (PPP) in light transmission aggregometry (LTA). Values are plotted with respect to sample order in the exam.

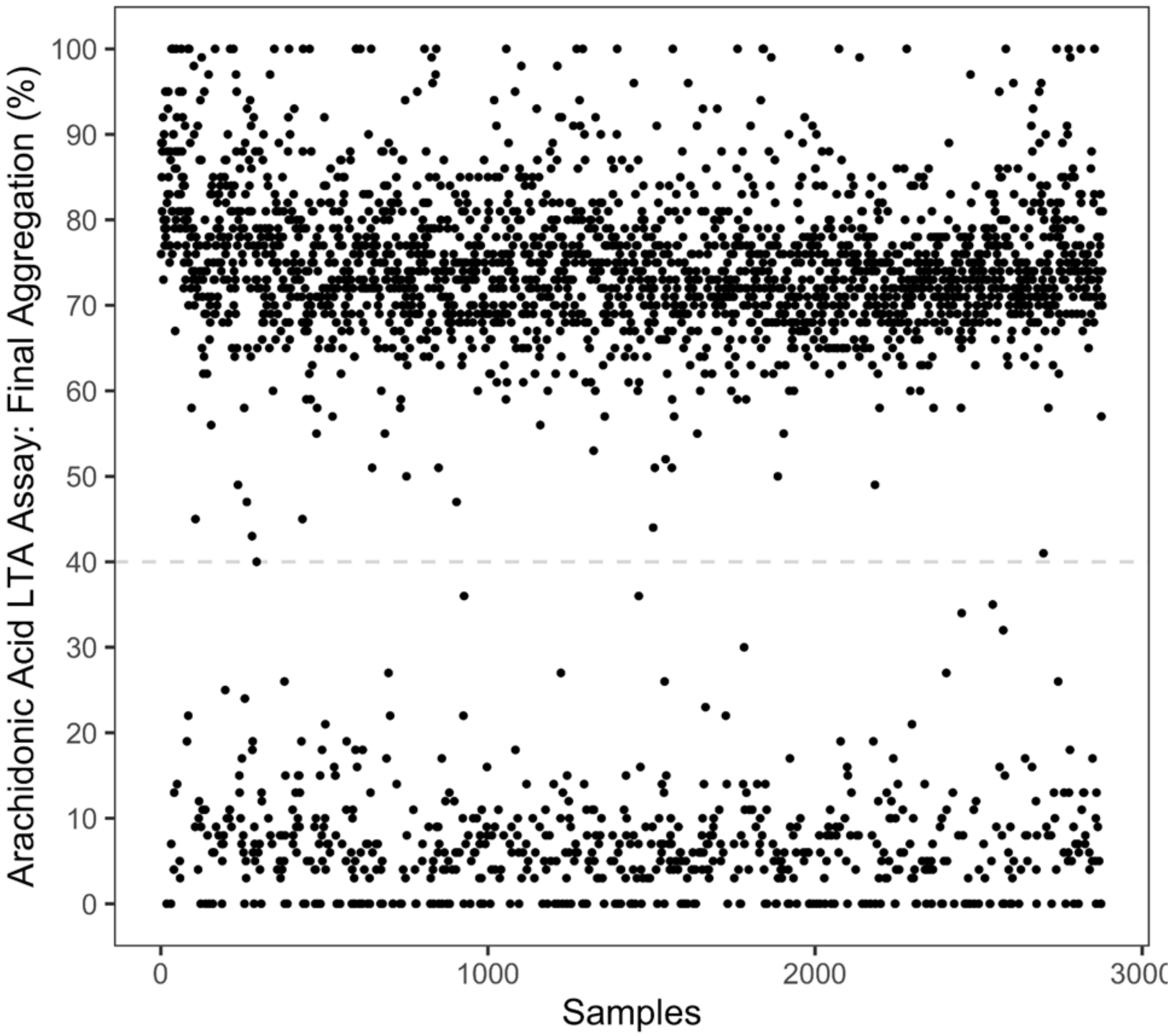

Supplement: Supplementary file 2 — Fig S1 [file CPT-111-909-s002.pdf]
